# On the Internal Use of Nitras Argenti, Combined with the Shower Bath, in Chorea Sancti-Viti

**Published:** 1815-04

**Authors:** Francis Edmund Franklyn

**Affiliations:** Lincoln


### *For the Medical and Physical Journal.*

### *On the Internal Use of Nitras Argenti, combined with the Shower Bath, in Chorea Sancti-Viti;* by Mr. F. E. Franklyn.

THE daughter of Mrs. Atkinson, of this city, aged thirteen years, applied to me for relief in a case of Chorea; and, having been previously discharged from the Infirmary as an incurable patient, I felt not a little interest in minutely examining the progress of the disease. On the 1st of September 1813, I was sent for, at a time when I found her affected with involuntary motions of the muscles of the face, producing great alteration in the features; a constant playing with her fingers, turning them in various forms, so that at times she was totally incapable of feeding herself; with great imbecility of the mental powers; and frequent fits of laughing and crying, without any evident cause. The complaint had been attended by a physician of this place for some time without any marked alterations; and, finding that she was prevented from making herself useful, she felt anxious to try still further. On inquiry, I found no natural evacuation had been procured for three days. I immediately prescribed

℞. Hydr. Submuriat. gr. iv.
Pulv. Jalapæ Com. gr. xij.

and, on the following morning, I had the satisfaction to find the bowels were fully emptied of their contents. The fæces, on examination, were found to be unnatural in colour, consistence, and smell, as they are often met with after a long remora,—the consequence of obstinate and protracted costiveness. Her strength was much impaired, but, her appetite continuing good, I now thought I might with some care venture on trying the internal use of Nitras Argenti, having heard of its success before. I prescribed as follows:

℞. Argenti Nitratis, ℈j.

Sapon. Alicant, ʒj.

Mucil. q. s. ut fiat massa divid. in Pilul. xl. capiat ij. ter in die; and by these means my patient continued to take three grains daily for a fortnight, without any unpleasant effects, which might have been expected from so deleterious an article. This powerful agent speedily excited an increased stimulus on the nervous system, so as to cut short the paroxysm of convulsions; and, in order to promote a still stronger impression, I desired my patient to have a bucket of water poured on her head every morning for a week, which had the decided effect of restoring her emaciation; yet, though I found cold affusion of benefit in this case, I cannot

I cannot be supposed to say that it will always answer. Dr. Currie, in his very valuable Reports, speaks of cold water as of inferior efficacy in this disorder, he having frequently tried it, but never found it of any service. The stimulating action of cold, though short in duration, is powerful in degree. In the torpor of convulsion, when weaker stimuli are unperceived, the affusion of cold water on the naked body will often excite the dormant sensibility, and introduce a new action throughout the nervous system.

FRANCIS EDMUND FRANKLYN.

*Lincoln; Feb.* 9, 1815.

---

*For the Medical and Physical Journal.*

